# Breaking the sound barrier: global monoclonal antibody pharmacoequity

**DOI:** 10.3389/fped.2026.1845146

**Published:** 2026-06-23

**Authors:** Huub C. Gelderblom, Glenda Gray, Brian Kelley

**Affiliations:** 1 Fred Hutch Cancer Center, Vaccine and Infectious Disease Division, Seattle, WA, United States; 2 South African Medical Research Council, Cape Town, South Africa; 3 Infectious Disease and Oncology Research Institute, University of Witwatersrand, Johannesburg, South Africa; 4 BK Biopharma Consulting, LLC, Burlingame, CA, United States

**Keywords:** access to medicines, biologics, disease prevention, essential medicines, monoclonal antibodies, pharmacoequity, RSV (respiratory syncytial virus) prophylaxis, volume catalyst

## Abstract

Monoclonal antibodies represent one of biomedicine's greatest successes, yet global access remains profoundly inequitable, with low- and middle-income countries bearing the brunt of limited access. This perspective proposes that long-acting infectious disease mAbs, starting with WHO-recommended nirsevimab for RSV prevention, could catalyze transformation by connecting with existing WHO Essential Medicines List mAbs. Picture two static half-circles: WHO Essential Medicines List mAbs with proven value but limited access, and infectious disease prevention mAbs with transformative potential. Connected, they form a complete wheel capable of rolling toward the aspirations of global pharmacoequity. HIV antiretroviral therapy demonstrates precedent—costs declined 100-fold through political mobilization, not technological breakthroughs.

## 1 Introduction: the static half-circles

Monoclonal antibodies (mAbs) represent one of biomedicine's greatest successes—yet global access remains profoundly inequitable. While more than 130 mAbs are approved in the United States (1), only sixteen appear on the current WHO Essential Medicines List (Table 1), and billions lack access to these life-saving drugs (2). The long-acting infectious disease mAb nirsevimab for RSV prevention when combined with the existing WHO Essential Medicines List mAbs create a critical mass that could catalyze improved access to mAbs in LMICs. As additional infectious disease mAbs for malaria and HIV gain approval, this combined effort could grow like a snowball.

**Table 1:** Who 2025 essential medicines list mAbs and RSV mAbs: global unmet need estimate.

mAb	Target	Year approved	EML section & indications	Annual tonnage for unmet need (tons)**
Cancer				
blinatumomab (Blincyto)^a^	Anti-CD19	2014	8.2.3 B-cell acute lymphoblastic leukemia	0.001
rituximab (Rituxan/MabThera)^a^	Anti-CD20	1997	5.1.2 multiple sclerosis 8.2.2 Burkitt lymphoma, diffuse large B-cell lymphoma, chronic lymphocytic leukemia, follicular lymphoma	1.1
trastuzumab (Herceptin)	HER2 receptor	1998	8.2.2 HER2-positive breast cancer	2.8
nivolumab (Opdivo)	PD-1 checkpoint	2014	8.2.3 metastatic melanoma	0.2
pembrolizumab (Keytruda)	PD-1 checkpoint	2014	8.2.3 metastatic cervical cancer, metastatic colorectal cancer, metastatic non-small cell lung cancer, metastatic melanoma	0.6
atezolizumab (Tecentriq)	PD-L1 checkpoint	2016	8.2.3 metastatic non-small cell lung cancer	3
cemiplimab (Libtayo)	PD-1 checkpoint	2018	8.2.3 metastatic non-small cell lung cancer	0.9
Hematology				
emicizumab (Hemlibra)^a^	Factor IX-a & X	2017	10.2 hemophilia A	1
Inflammatory disease				
infliximab (Remicade)^a^	TNF-alpha*	1998	8.1 immunomodulators for non-malignant disease 13.4 medicines affecting skin differentiation and proliferation 29.3 medicines for juvenile joint diseases	1.1
adalimumab (Humira)^a^	TNF-alpha*	2002	8.1 immunomodulators for non-malignant disease 13.4 medicines affecting skin differentiation and proliferation 29.3 medicines for juvenile joint diseases	6.5
certolizumab pegol (Cimzia)^a^	TNF-alpha*	2008	8.1 immunomodulators for non-malignant disease 13.4 medicines affecting skin differentiation and proliferation 29.3 medicines for juvenile joint diseases	2.7
golimumab (Simponi)	TNF-alpha*	2009	8.1 immunomodulators for non-malignant disease 29.3 medicines for juvenile joint diseases	0.3
ustekinumab (Stelara)^a^	IL-12 & IL-23 antagonist	2023	13.4 medicines affecting skin differentiation and proliferation	1.1
Infectious disease				
Anti-rabies mAbs (Rabishield)^a^	Rabies virus		19.2 sera, immunoglobulins and monoclonal antibodies	1.1
ansuvimab-zykl (Ebanga)^a^	Ebola virus	2023	6.7 medicines for Ebola virus disease	0.004
atoltivimab, maftivimab, odesivimab (Inmazeb)^a^	Ebola virus	2023	6.7 medicines for Ebola virus disease	0.012
Nirsevimab (Beyfortus)***	RSV	2023	Not on EML	7-14
Clesrovimab (Enflonsia)	RSV	2025	Not on EML	***
Total				30-37

a Also listed on the Essential Medicines List for Children (EMLc).

* Several mAbs are interchangeable, this is taken into account in the calculations.

** Order-of-magnitude estimates based on global disease burden and standard dosing regimens, assuming ∼100 LMIC without access, 70 kg body weight, 1.7 m^2^ surface area.

*** Nirsevimab is the WHO-recommended long-acting RSV mAb for all infants (single dose protection). Clesrovimab represents an alternative long-acting option. The math for global access remains similar regardless of which RSV mAb is used.

**Figure 1:**
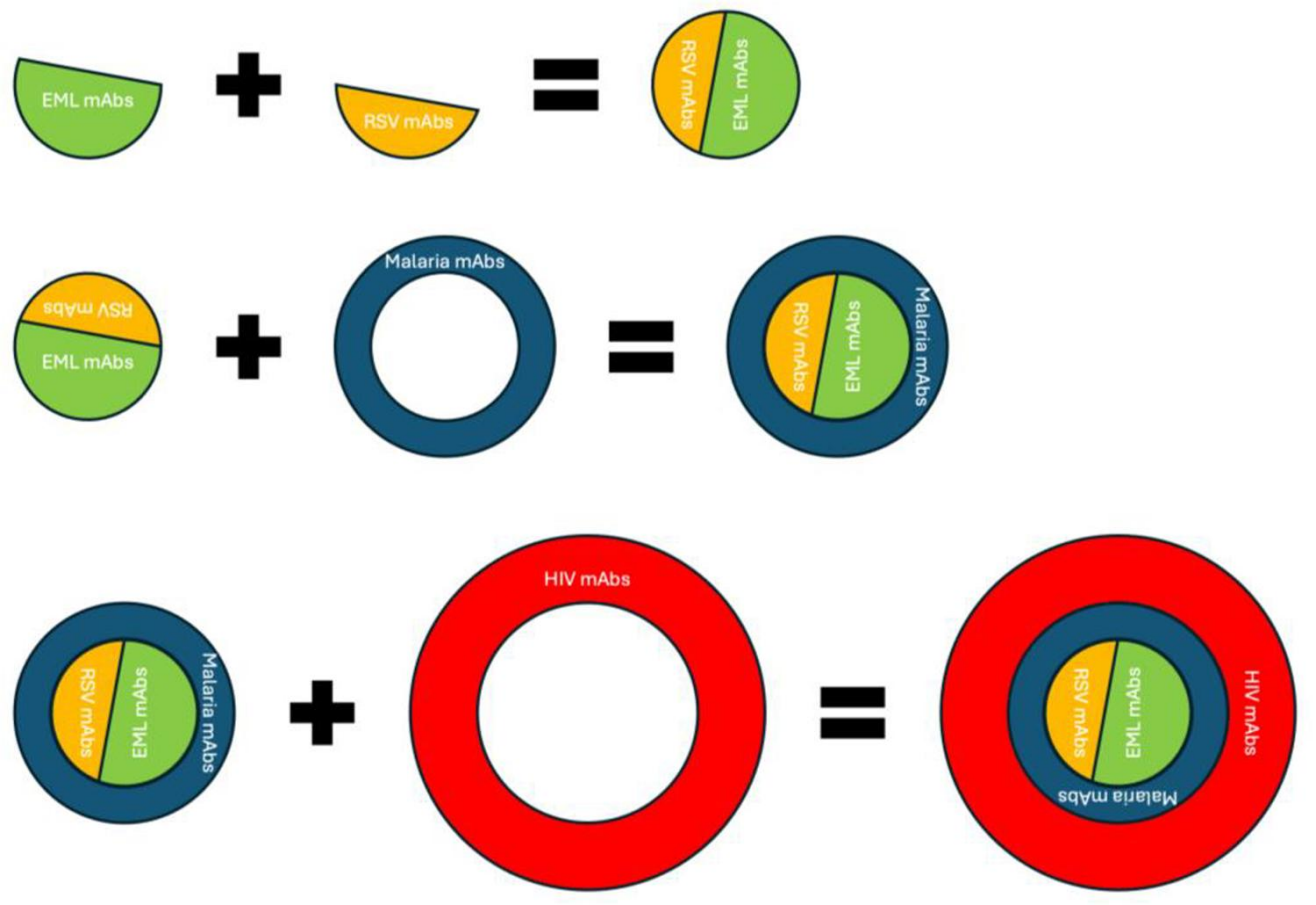
Combining half circles to create momentum for all mAbs. EML, WHO Essential Medicines List.

Picture two half-circles lying motionless on the ground (Figure 1). The first represents the monoclonal antibodies on the WHO Essential Medicines List—rituximab (approved 1997), trastuzumab (1998), infliximab (1998), and others that transformed cancer and autoimmune disease treatment in high-income countries. Despite being available in high-income countries for 15-30 years, these therapeutic breakthroughs remain largely inaccessible to billions of people in approximately 100 low- and middle-income countries (LMICs). This half-circle, representing roughly 23 metric tons of annual unmet global need, sits static—powerful in potential but unable to generate momentum for systemic change (2). Is the inertia driven by cost of goods or that drug companies simply are not interested in those markets because of poor ROIs?

The second half-circle represents long-acting infectious disease prevention mAbs, starting with nirsevimab for prevention of respiratory syncytial virus (RSV). Nirsevimab when administered at birth or at the start of RSV season is highly effective in preventing severe RSV disease (3–6).

## 2 The wheel begins to roll: infectious disease mAbs as infrastructure

The WHO recommendation for nirsevimab for RSV prevention represents both an unprecedented public health opportunity and the key to unlocking systemic transformation (2). The two half-circles represent complementary volumes and populations. The first half-circle—WHO Essential Medicines List mAbs—requires 23 metric tons annually but serves smaller, targeted populations with cancer and autoimmune diseases. The second half-circle—long-acting infectious disease prevention mAbs starting with nirsevimab—requires 7-14 metric tons annually but reaches 140 million infants globally. Like the segments and peel of an orange, these complementary volumes create a complete, integrated whole when connected. The combination of RSV and WHO Essential Medicines List mAbs requires 30-37 metric tons, the production scale needed to make both economically viable.

The fragmented, disease-specific approach to mAb access is exemplified by pembrolizumab in India, where despite the country's position as a global biosimilar manufacturing hub, less than 1% of eligible patients access this life-saving therapy at ∼$6,000 per cycle, because individual disease markets lack the volume to justify dedicated manufacturing that could reduce costs to $50-100/gram (7). This demonstrates why WHO Essential Medicines List mAbs cannot achieve momentum independently—even powerful cancer immunotherapies remain trapped in high-cost, low-access paradigms when treated as isolated markets rather than components of a unified therapeutic class.

This requires expanded manufacturing capacity—whether through scaling existing facilities or establishing new production sites closer to demand—plus temperature-controlled distribution networks, integration with maternal-child health programs, harmonized regulatory pathways, and community demand generation. Once this RSV + WHO Essential Medicines List connection is established and working, the same manufacturing and delivery systems can absorb malaria mAbs, HIV mAbs, and eventually grow to accommodate the full infectious disease portfolio as it gains approval: malaria prevention for 110 million children, HIV pre-exposure prophylaxis for 20-100 million children and adults, and HIV treatment for up to 40 million people living with HIV (8–11).

The predictability is equally transformative. Birth cohorts, malaria-endemic populations, HIV incidence and prevalence, cancer incidence change slowly year-to-year, enabling long-term contracts and planning.

## 3 The historical precedent: HIV treatment's 100-fold cost reduction

The history of HIV antiretroviral therapy (ART) provides a crucial precedent demonstrating that barriers to affordable biologics are primarily structural and political rather than technical. Between 2000 and 2008, ART costs in LMICs declined from approximately $10,000 per person-year to less than $100—a 100-fold reduction achieved in less than eight years not through manufacturing breakthroughs, but through political mobilization (24).

Many actors and events created a perfect storm of political pressure, intellectual property flexibility, guaranteed procurement volumes, and manufacturing capacity. The 13th International AIDS Conference in Durban, South Africa in 2000 proved pivotal—where 11-year-old Nkosi Johnson, born with HIV, spoke to delegates about the human cost of unequal access. The Treatment Action Campaign in South Africa, the Doha Declaration on TRIPS and Public Health (2001), PEPFAR and Global Fund establishment (2003), and Indian generic manufacturing scale-up transformed global treatment access. The same molecules—manufactured using identical or similar processes—cost 40-fold more in high-income countries ($3,000) than LMICs ($75) in 2024, a differential sustained for two decades through structural barriers (24).

This comparison reveals that monoclonal antibodies have followed the slowest learning curve among major technologies over nearly four decades, declining only 33-fold despite innovations from establishment of Chinese Hamster Ovary cell lines platforms and initial scaling (era 1, 1975-1998) to blockbuster mAb commercialization (era 2, 1999-2019), to unprecedented clinical development and scale-up to >30 tons annual production of COVID-19 mAbs (era 3, 2020-2023), to further optimization and expansion in the present era 4 (12). When political intervention created alternative market dynamics for ART, costs declined faster than mAb manufacturing optimization achieved through pure technological advancement.

Unlike complex antibody-drug conjugates, these mAbs are relatively simple—nirsevimab is an IgG1 antibody with YTE (3 amino acid mutation in the Fc part to extend the half-life by about 3-fold) as the only mutation. The persistence of high costs for such straightforward molecules highlights how structural barriers, not technical complexity, drive pricing.

This fragmentation persists today with devastating consequences. WHO recommends nirsevimab for all infants globally to prevent RSV disease, yet the same organization has not prequalified the medicine and excludes it from the Essential Medicines List (25). This bureaucratic dysfunction prevents UNICEF procurement while 100,000 children die annually from preventable RSV disease in LMICs without access to nirsevimab—history repeating itself with tragic precision (13). Just as HIV treatments existed in the 1990s but structural barriers delayed access for a decade, we now witness the same pattern with pediatric RSV prevention.

The lesson is clear: innovation alone is necessary but insufficient. Structural barriers—intellectual property regimes, procurement fragmentation, manufacturer market power—often determine access more than scientific breakthroughs.

## 4 The second wheel: eight segments for pharmacoequity

**Figure 2:**
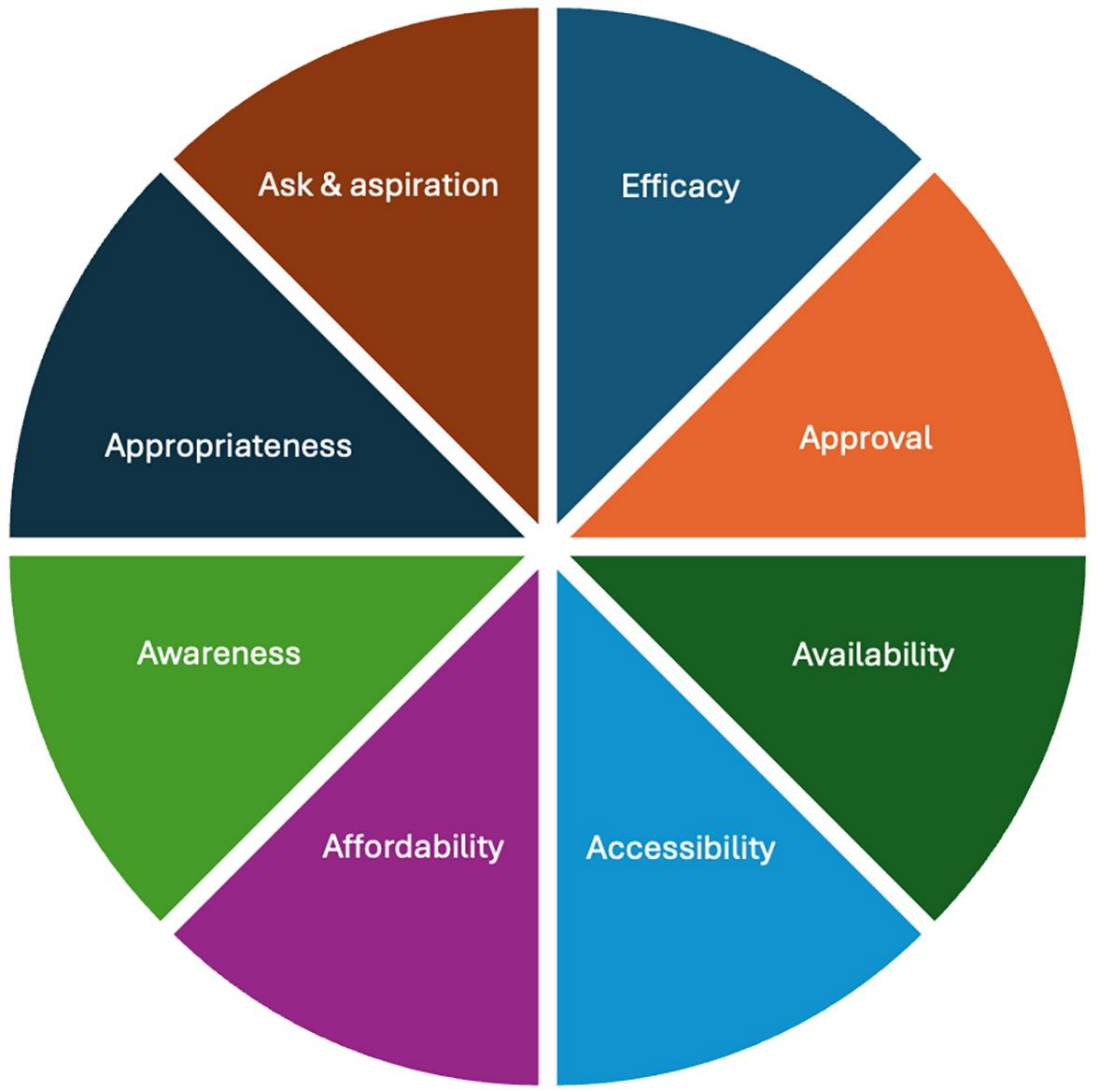
The wheel of pharmacoequity: From efficacy in clinical trials to effectiveness in the real world.

Moving from efficacy in a clinical trial to global population-level effectiveness requires addressing eight elements (segments, Figure 2) in parallel: **(1) Efficacy** demonstration in trials, **(2) Regulatory approval** through harmonized pathways, **(3) Availability** via technology transfer and local manufacturing, **(4) Accessibility** through delivery infrastructure, **(5) Affordability** via pooled procurement and TRIPS flexibilities, **(6) Awareness** among providers and patients, **(7) Appropriateness** for local contexts, and **(8) Ask/Aspiration/Political will**—the fundamental demand that life-saving interventions should be universally accessible regardless of geography or income (14) and the political will with concomitant budget allocation either at a government level or by global procurement agencies. The critical insight is parallel rather than sequential implementation. Engage the Medicines Patent Pool and generic manufacturers during Phase 3 trials. Negotiate technology transfer before licensure.

The “Aspiration” element may prove most fundamental. For decades, people in low-income countries were told antiretrovirals were too expensive, too complex to work outside high-income settings. This self-fulfilling prophecy that led to 10-12 million deaths (15) was shattered by among others the Treatment Action Campaign's assertion of equal right to life-saving treatment and studies showing that adherence to ART was better in rural Uganda than in the US (16, 17). The same assertion is required for mAbs: children in Mali and Kenya have identical rights to RSV protection as children in London and New York. More than 100,000 children die of RSV each year in LMIC that do not have access to nirsevimab, compared to less than 500 (before introduction of nirsevimab) in high-income countries that now have access (13). Infants and toddlers can't advocate for themselves. We have to do that.

## 5 Expanded infectious diseases scope: mAbs for antimicrobial resistance, influenza prevention, pandemic preparedness capacity

Expanding the wheel’s momentum, monoclonal antibodies targeting influenza, antimicrobial resistance (AMR) and pandemic preparedness represent the next frontier for integrated global access.

The COVID-19 pandemic demonstrated both the potential and limitations of mAb responses. While therapeutic mAbs like bebtelovimab provided crucial treatment options, production delays and strain-specific targeting limited global impact. The integrated manufacturing infrastructure proposed here could enable rapid pandemic mAb deployment, with established production capacity, regulatory pathways, and delivery networks ready for activation during emerging infectious disease outbreaks (12, 18).

Seasonal influenza causes 290,000-650,000 deaths annually, with disproportionate impact in LMICs (19). Monoclonal antibodies targeting influenza are in clinical development (20, 21).

AMR causes approximately 1.27 million deaths annually, with particularly severe impact in LMICs where healthcare-associated infections encounter limited treatment options. mAbs against *Staphylococcus aureus* and *Clostridium difficile* are in advanced clinical development (22). Unlike traditional antibiotics facing inevitable resistance, mAbs can target virulence factors, biofilms, and immune evasion mechanisms while potentially maintaining efficacy longer. For 50 million annual patients requiring AMR prophylaxis (100-500 mg doses), approximately 5-25 metric tons would be needed—adding meaningful volume to infectious disease mAb infrastructure while addressing one of modern medicine's greatest threats.

## 6 Era 5: is there a mAb for that?

Following the four eras of mAb development outlined above we could now enter era 5, characterized by cost-driven accessibility and therapeutic expansion. The rolling wheel unlocks therapeutic possibilities currently constrained by cost and fragmentation. With manufacturing costs decreasing further, mAbs become viable for indications beyond traditional oncology and autoimmune diseases. AMR, influenza prevention, migraine prevention, allergies, Alzheimer's disease, substance use disorders including fentanyl addiction—countless therapeutic targets remain unexplored because current mAb pricing makes development economically unfeasible or clinically inaccessible. The integrated manufacturing and delivery infrastructure creates the foundation for systematic exploration of the full therapeutic potential of monoclonal antibodies (21, 23).

The One Health approach reveals an additional volume catalyst: veterinary mAbs. With 900 million dogs, 600 million cats, and billions of livestock globally, animal health represents massive untapped demand. Existing veterinary mAbs like lokivetmab (Cytopoint, 2017) for atopic dermatitis, bedinvetmab (Librela, 2021) for canine osteoarthritis, and frunevetmab (Solensia, 2022) for feline pain demonstrate market viability. The same manufacturing infrastructure serving human infectious disease mAbs could simultaneously produce veterinary products, creating additional volume to drive costs down while serving both human and animal health needs.

## 7 The rolling wheels: integrated delivery vision

Once the wheel begins rolling, integrated delivery becomes possible. Maternal-child health programs delivering nirsevimab can simultaneously distribute WHO Essential Medicines List mAbs. The same cold chain infrastructure, community health workers, and regulatory pathways serve multiple products. This integration transforms episodic, disease-specific interventions into comprehensive pharmacoequity infrastructure. The wheel's momentum creates economies of scale—where delivering multiple mAbs together costs less than delivering each separately.

## 8 Breaking the sound barrier

“Breaking the sound barrier” represents the breakthrough moment when accumulated momentum overcomes systemic resistance. Like an aircraft breaking the sound barrier or encountering turbulence, the combined wheel of WHO Essential Medicines List mAbs and infectious disease prevention faces ups and downs, challenges and obstacles, as it travels the long and winding road to pharmacoequity. Individual efforts—whether rituximab access programs or standalone RSV campaigns—encounter turbulence and often stall. But when sufficient momentum builds through integrated volume, manufacturing scale, and delivery infrastructure, the wheel can break through to sustained, equitable access.

The barrier is not technical—we know how to manufacture these mAbs at scale. The barrier is structural: fragmented markets, intellectual property constraints, procurement silos, and regulatory inefficiencies. Once broken, the barrier reveals clear skies ahead: predictable demand, streamlined production, integrated delivery, and ultimately, pharmacoequity as the foundation rather than the exception.

## 9 Challenges and limitations

This vision faces significant challenges. Intellectual property regimes may limit generic competition. Regulatory harmonization requires unprecedented coordination. Cold chain infrastructure demands substantial investment. Political commitment must sustain across electoral cycles. Most critically, the approach requires viewing mAbs as a class of drugs rather than individual products—a shift from disease-specific silos to integrated pharmacoequity infrastructure.

Yet these challenges reflect structural barriers, not technical impossibilities. The HIV treatment precedent demonstrates that political mobilization can overcome seemingly insurmountable obstacles when the imperative is clear.

## 10. Conclusion: the wheels in motion

The two half-circles lie before us: WHO Essential Medicines List mAbs with proven therapeutic value but limited access, and infectious disease prevention mAbs with transformative potential but uncertain implementation. Separately, they remain static. Together, they form a complete wheel capable of rolling toward global pharmacoequity.

An African proverb reminds us: “If you want to go fast, go alone. If you want to go far, go together.” Individual mAb access programs—rituximab biosimilars, standalone RSV campaigns, disease-specific initiatives—may achieve rapid initial progress. But sustainable pharmacoequity requires going together: connecting WHO Essential Medicines List mAbs with infectious disease prevention, integrating manufacturing with delivery, aligning regulatory pathways across therapeutic areas. This collaborative approach may seem slower initially, but it builds the momentum needed to go far—toward a future where pharmacoequity is the foundation, not the exception.

The wheel can start rolling today by connecting nirsevimab for RSV with existing WHO Essential Medicines List mAb initiatives. As malaria and HIV mAbs gain approval, the wheel grows like a snowball, building momentum that becomes increasingly difficult to stop. Manufacturing investments become profitable. Delivery infrastructure becomes sustainable. Pharmacoequity becomes inevitable.

Thirty years after the first therapeutic mAb, we stand at an inflection point. We can continue the fragmented, fast-but-limited approach that has left billions without access to essential medicines—an approach that, for mAbs, has proven neither fast nor effective despite decades of effort. Going together may be the only viable path to mAb pharmacoequity. Together, we can connect the two half-circles and get the wheel rolling toward a future where place of birth no longer determines access to these essential medicines.

## Data Availability

Publicly available datasets were analyzed in this study. This data can be found here: https://www.who.int/data/sets/health-inequality-monitor-dataset#ihme-gbd https://www.who.int/publications/i/item/B09474.
